# Influence of rice-husk biochar and *Bacillus pumilus* strain TUAT-1 on yield, biomass production, and nutrient uptake in two forage rice genotypes

**DOI:** 10.1371/journal.pone.0220236

**Published:** 2019-07-31

**Authors:** Khin Thuzar Win, Keiki Okazaki, Taiichiro Ookawa, Tadashi Yokoyama, Yoshinari Ohwaki

**Affiliations:** 1 Central Region Agricultural Research Center, National Agriculture and Food Research Organization Tsukuba, Ibaraki, Japan; 2 Tokyo University of Agriculture and Technology, Fuchu, Tokyo, Japan; RMIT University, AUSTRALIA

## Abstract

Biochar is widely used as a soil amendment to increase crop yields. However, the impact of the interaction between the biochar and microbial inoculants (e.g., biofertilizer) on plant nutrient uptake and yield in forage rice is not fully understood. A greenhouse study was conducted to evaluate the synergistic effects of rice-husk biochar and *Bacillus pumilus* strain TUAT-1 biofertilizer application on growth, yield, and nutrient uptake in two forage rice genotypes; Fukuhibiki and the newly bred line, LTAT-29. Positive effects of biochar and biofertilizer, alone or in a combination, on growth traits, nutrient uptake, and yield components were dependent on the rice genotypes. Biochar and TUAT-1 biofertilizer influenced the overall growth of plants positively and increased straw and above-ground biomass in both genotypes. However, although biochar application significantly increased grain yield in LTAT-29, this was not the case in Fukuhibiki. Biochar and TUAT-1 biofertilizer, either alone or combined, significantly affected plant nutrient uptake but the effect largely depended on rice genotype. Results of this study indicate that biochar amendment and TUAT-1 biofertilizer can enhance forage rice productivity depending on genotypes, and therefore, there is a need to consider plant genetic composition when evaluating the potential for crop response to these soil amendments before application on a commercial scale.

## Introduction

Rice (*Oryza sativa* L.) is the staple food for more than 50% of the world’s population, particularly in Asia. In Japan, it is also used for multiple purposes, such as flour, livestock feed (including whole-crop silage), and biofuel [1]; rice plants, used for soil amendment, can maintain soil fertility. Currently, forage rice production is being promoted by the government in Japan since the cultivation of forage crop in excess paddy fields is considered a promising way to enhance feed supply. In addition, approximately 75% of the domestic demand of feed for livestock is dependent on imports from overseas [2]. To increase the percentage of self-sufficient food supply and improve the domestic demand of livestock feed in Japan, production of forage rice is increasing in Japan quickly since the last decade [3]. To meet these demands, high amounts of N fertilizer are applied to obtain a high yield of rice. However, excessive N input will lead to an inefficient use of N and large N losses to the environment, adversely affecting air and water quality, biodiversity, and human health [4].

One creative idea to assist in the transition toward sustainability by improving agricultural systems and resource management is the pyrolysis of crop biomass bi-product (biochar) and its incorporation into soil systems [5]. Biochar has been widely recognized for its property to cause beneficial soil amendment by improving soil physical, chemical, and biological properties [6–8] as well as in retaining nutrients, thereby enhancing plant growth [9]. Because biochar contains organic matter and nutrients, its addition increased the soil pH, electric conductivity (EC), organic carbon (C), total nitrogen, available phosphorus (P), and cation-exchange capacity (CEC) [6–8]. Recently, biochar has been shown as a soil conditioner that can enhance fertilizer N uptake by rice, resulting increase productivity [10], and suppress leaching and improving plant N use [11–12].

For rice-based farming systems, rice-husk biochar is considered to be one of the most cost-effective biochars and has been used for a long time [13]. Recently, Koyama *et al*. [14] and Asai *et al*. [15] also found a positive effect of biochar on rice production, emphasizing the dependence on soil fertility and fertilizer management. Thus, the integration of biochar in agricultural systems has been identified as a promising strategy to enhance agricultural yield and, via the potential for carbon sequestration, as a means to mitigate the negative impacts of agricultural production [6, 7], while simultaneously reducing N_2_O [16].

Use of efficient plant growth-promoting bacteria (PGPB) inoculant biofertilizer would be another sustainable route to a better performance, in terms of soil properties and crop yield. Plant growth-promoting rhizobacteria (PGPR) are rhizosphere-inhabiting bacteria that have a positive influence on plant growth and development likely owing to bacterial production of plant growth regulators such as auxins, gibberellins, and cytokinins [17, 18]. Several root-colonizing *Bacillus* species are well known for their growth-promoting effect [19]. Previous studies have indicated that *Bacillus pumilus* strain TUAT-1 (hereafter referred to as TUAT-1) has been found to increase growth in forage rice variety Leaf Star [20]; in the rice variety Koshihikari [21]; and in other crops, including komatsuna, mustard, and radish [22], mainly owing to its potential to increase nutrient uptake by enhancing root growth. Although there is vast amount of data evidence on growth enhancement of TUAT1, to our knowledge, a consistent positive interaction between the bacteria and plant genotype remains unclear. Furthermore, generally, bacterial inoculation improves plant growth and rice yield but not uniformly because the beneficial effect of PGPB on crop growth varies considerably depending on soil nutrition, plant species, inert material quality, inoculant density, and environmental condition.

It is well reported that incorporation of biochar into soil alters the soil microbial community and diversity owing to its high surface area, porous nature, and capacity to act as a medium for microorganisms [7]. It is postulated that biochar may promote survival of target inoculated bacteria after inoculation and may act as a stable artificial shelter for inoculated bacteria. If added with biochar, they may not only result in an enhancement of crop yield owing to its great potential as available nutrient but also help in preventing fertilizer run off, leaching, retaining moisture, and acting as a carrier for PGPR. On one hand, TUAT-1 inoculation was reported to enhance root growth presumably owing to phytohormone production and consequently improved nutrient and water uptake. Thus, understanding interactions between biochar and biofertilizer is of great importance for improving rice yield and nutrient uptake in two different forage rice genotypes. Our hypothesis was to address the synergistic effects of biochar and TUAT-1 biofertilizer on promoting of the growth, yield, and nutrient uptake of two forage rice genotypes. This work may contribute to developing application strategies with biochar and TUAT-1 for dealing with the yield-promoting potential in the field.

In addition, reports on the effects of biochar on different plant species and cultivars within a species have rarely reported on the underlying basis of biochar-mediated plant growth promotion. Although biochars have been extensively documented for their positive impact on plant growth and development, by altering soil properties and the bioavailability of nutrients [9], the functional response of the interaction between PGPB and biochar amendments on plant genotypes is not well understood. Here we investigated the genotype-specific effects of biochar on two forage rice genotypes in combination with TUAT-1 biofertilizer.

In this context, the objective of this study was to evaluate the effect of biochar and TUAT-1 biofertilizer individually and in combination on plant growth, physiological performance, yield, yield components, and nutrient uptake of two forage rice genotypes.

## Materials and methods

### Soil preparation and analysis

For experiments conducted in pots, the soil was collected from a paddy field at the National Agricultural and Food Research Organization, Yawara experimental site, Tsukubamirai city, Japan (36°00′N, 140°01′E). Before the introduction of water for field planting, top soil (depth 0–15 cm) samples were collected at the three vertices of an equilateral triangle (side length 10 m) marked out in the field, which were mixed together to give a composite soil. The soils were air dried and passed through a 2.0-mm sieve. In the Yawara field, the soil is classified as a fine-textured Haplic Gray Lowland soil [23] that corresponds to Gleyic Fluvisols in WRB [24] and Typic Fluvaquent in the USDA soil taxonomy [25]. The soil physicochemical properties before rice cultivation was characterized by pH (1:2.5 soil: water ratio) of 6.18, electrical conductivity (EC) of 0.1 dS m^−1^, CEC of 16.2 cmol_c_ kg^-1^, exchangeable K of 460 mg kg^−1^, exchangeable Ca of 2780 mg kg^−1^, exchangeable Mg of 570 mg kg^−1^, available N of 90 mg kg^−1^, available P (Truog) of 75 mg kg^−1^, humus of 5.8%, and P absorption capacity of 13000 (P_2_O_5_ mg kg^−1^). Phosphorous absorption was determined following the procedure of Nanzyo et al. [26].

### Rice varieties and nursery preparation

The rice varieties used in this study were Fukuhibiki, a mutant cultivar with high grain yield, excellent plant qualities, and high tolerance to lodging, and LTAT-29 (Monster rice-1) having a super high grain yielding ability, long, and thick culms, and large panicles. Fukuhibiki was developed from the progeny of Kochihibiki/82Y5-31 (Ouu 316), and LTAT-29 was a hybrid line between the progeny of TUAT (Akenohoshi × Takanari) and TULT (Leaf Star × Takanari). Both forage rice genotypes used in this study are used as feed, and the grains are used as concentrated feed for domestic fowl and animals. Seedlings were prepared by soaking the seeds in tap water for 3 days at 25°C after sterilizing in hot water (60°C) for 10 min, followed by air-draining and incubation for 1 day at 25°C until germination. Pre-germinated seeds were uniformly broadcast in a nursery tray (30 × 60 cm) on April 25, 2017. Commercial rice nursery soil (Kanuma A, JA) was used for raising the seedlings. Before sowing the pre-germinated seeds, nursery soil medium was thoroughly mixed with granular biofertilizer with or without TUAT-1 (5% of the soil weight). The population density of TUAT-1 *Bacillus* cells in the biofertilizer was approximately 1.2 × 10^7^ colony forming units (CFU) g^−1^ [21]. Treatments were arranged in a completely randomized design with four replicates.

The nursery seedlings were raised under greenhouse conditions for 21 days. Thirty 21-day-old seedlings were randomly selected from three locations in each nursery tray, and the following data were recorded: shoot length, and shoot and root fresh and dry weight. For the purpose of root colonization, we used a TUAT-1 strain with antibiotic resistance to streptomycin and rifampicin [27]. Colonization was examined using 15 plants randomly sampled and combined (Table 1). The roots were washed thoroughly in sterilized Milli-Q water. One gram of fresh root was crushed under sterile conditions in 10 ml of phosphate buffer, using a pestle and mortar. The suspension was serially diluted and spread on TSA medium with 100 mg L^−1^ of streptomycin and rifampicin [27]. The plates were incubated at 28°C for 24 h before determining the CFU mL^−1^. The total numbers of bacteria were calculated as the number of CFU per gram of root fresh weight after 2-day incubation. Agronomic characteristics and colonization results for the seedlings are described in Table 1.

**Table 1:** Effect of inoculation of TUAT-1 on morphological parameters in two rice genotypes grown in nursery seedling box at 21 days after inoculation.

		Shoot length (cm)	Fresh weight (mg)		Dry weight(mg)		CFU/g fresh root
			shoot	root	shoot	root	
**Fukuhibiki**	**Control**	22.3 ± 0.79^c^	167.3 ± 12.22^c^	77.1 ± 8.89^a^	25.8 ± 3.66^a^	11.3 ± 1.30^a^	-
	**Bio (TUAT-1)**	24.8 ± 0.36^a^	186.2 ± 16.67^b^	82.2 ± 5.39^a^	30.6 ± 3.68^a^	10.9 ± 0.77^a^	3.6 × 10^3^
**LTAT-29**	**Control**	23.7 ± 0.55^b^	189.8 ± 4.52^b^	81.3 ± 7.69^a^	28.7 ± 1.77^a^	11.6 ± 1.02^a^	-
	**Bio (TUAT-1)**	25.6 ± 0.57^a^	211.6 ± 2.78^a^	87.8 ± 12.87^a^	31.1 ± 1.92^a^	11.8 ± 1.01^a^	4.2 × 10^3^
**Analysis of variance**							
**Genotype (G)**		**	**	ns	ns	ns	
**Bio**		**	**	ns	ns	ns	
**G×Bio**		ns	ns	ns	ns	ns	

Mean ± SD followed by the same letter are not statistically significant using Fisher’s LSD test (p < 0.05).

** p < 0.01

ns = not significant at 0.05 level

### Experimental set-up and rice cultivation

Three 21-day-old seedlings with or without application of TUAT-1 biofertilizer in the nursery bed were transplanted into 1/5,000a Wagner pots (16 cm diameter, 20 cm height) containing 3.5 kg of paddy soil with or without biochar (Pros Co. Ltd., Nagano, Japan) whose physicochemical characteristics were previously described by Koyama *et al*. [14]; briefly, bulk density was 0.13 g cm^−3^, pH (H_2_O) 8.4, EC 35.8 dS m^−1^, total N 4.01 mg kg^−1^, total C 427 mg kg^−1^, Si 192,000 mg kg^−1^, K 8,710 mg kg^−1^, Ca 2,170 mg kg^−1^, and Mg 590 mg kg^−1^. The biochar was thoroughly mixed with the soil at the rate of 21 g kg^−1^ of soil. Nitrogen from slow release N fertilizer, LP40 0.5 g N, 0.5 g P (P_2_O_5_), and 0.5 g K (K_2_O) per pot were added as a basal fertilizer application. Upon transplanting, to gain deeper insights into the possible longer-term effects of inoculation into soils containing target inoculated bacteria, the seedlings were re-inoculated by soaking for 1 h in a suspension of *Bacillus pumilus* strain TUAT-1 (10^7^ CFU mL^−1^) or tap water. The experiment included four treatments: 1) control (no biofertilizer and no biochar, CT), 2) TUAT-1 biofertilizer only (Bio), 3) biochar only (BC), 4) TUAT-1 biofertilizer with biochar (Bio+BC). All treatments were tested on two forage rice genotypes (Fukuhibiki and LTAT-29). After transplanting, the pots were maintained under submerged conditions throughout the course of the experiments and actively aerated by physically disturbing and breaking up the soil surface once every three days. At the booting stage, top-dressing of N fertilizer at the rate of 0.3 g N pot^−1^ was conducted in all pots. Final drainage was conducted 10 days before harvest and the rice plants were harvested at the maturity stage (123 DAT). The daily mean air temperature in the glasshouse ranged from 25°C to 31°C during the experiment.

### Measurement of rice growth and physiological components

Plant height was measured from ground level to the tip of the longest leaf, and tiller number was counted at first tillering (2 WAT, weeks after transplanting), maximum tillering (6 WAT), booting, and heading stages. Rice physiological characteristics and chlorophyll content (SPAD value) were measured at 6 WAT. Measurements for the physiological characteristics were made on flag leaves. Measurements of net photosynthesis (Pn; μmol CO_2_ m^−2^ s^−1^) were monitored using a LICOR 6400 portable photosynthesis system (Lincoln, Nebraska, USA). During measurements, light intensity in the assimilation chamber was set to 1000 μmol m^-2^ s^−1^ and CO_2_ concentration to 400 μmol mol^−1^. These measurements were taken on a clear sunny day between 09:45 and 11:30 under saturated light conditions (solar radiation > 1200 μmol m^−2^ s^−1^).

### Definition of nitrogen use efficiency (NUE), nitrogen utilization efficiency (NUtE), nitrogen uptake efficiency (NUpE)

NUE is the product of NUpE and NUtE, where NUpE is the capacity of plant roots to acquire N from the soil and NUtE is the plant biomass productivity per unit of N uptake calculated following the protocol of Menz *et al*. [28].:

$$NUE\;(straw)=\frac{straw\;biomass\;(g)}{applied\;N\;in\;pot\;(g)}$$

$$NUE\;(grain)=\frac{grain\;weight\;(g)}{applied\;N\;in\;pot\;(g)}$$

$$NUtE\;(straw)=\frac{straw\;biomass\;(g)}{straw\;N\;uptake\;(g)}$$

$$NUtE\;(grain)=\frac{grain\;weight\;(g)}{grain\;N\;uptake\;(g)}$$

$$NUpE=\frac{above-ground\;N\;uptake\;(g)}{applied\;N\;in\;pot\;(g)}$$

### Measurement of macro- and micronutrient concentrations

For the quantification of ion present in the straw and grain, 20 mg of dried sample (powder) was digested in 300 μl of concentrated nitric acid (HNO_3_), incubated at 100°C for 3–4 h, cooled at room temperature for 1 h, and the volume made up to 10 ml with Milli-Q water [29]. Mineral elemental analysis of the plant extracts was determined by inductively coupled plasma–mass spectrometry (Agilent 7700X, USA).

### Post-harvest soil analysis

At the end of the experiments (after crop harvest), composite soil samples were collected from each pot, air dried, and sieved (2 mm). Soil samples were then stored in a cool and dry place until analyzed. Soil physicochemical characteristics were analyzed by Katakura Coop Agri Co., Ltd. (Tsuchiura, Japan), which are briefly listed below.

Soil pH and EC were measured in deionized water at a ratio of 1:5. Exchangeable base cations (K^+^, Mg^2+^, and Ca^2+^) in the soil were extracted with 1 M ammonium acetate at pH 7, and concentrations of these cations were determined using the Spectro-Flame-Photometer (SEP-4i, Fujihira, Tokyo, Japan) for K^+^, colorimetric determination of Mg^2+^ with xylidyblue [30], and the o-cresolphthalein complexone (oCPC) method for Ca^2+^ [31]. CEC was determined using the indophenol blue colorimetric method using the Spectro-Flame-Photometer (SEP-4i, Fujihira, Tokyo, Japan). Available phosphate was extracted in 0.002 N H_2_SO_4_ buffered with (NH_4_)_2_SO_4_ at pH 3.0 by the Troug method and was colorimetrically determined [32]. The ammonium (NH_4_^+^) and nitrate (NO_3_^−^) contents were extracted with 1 M KCl and determined by QuAAtro2-HR BL-TEC autoanalyzer. Soil available Si content was extracted by Na-acetate buffer (pH 4.0). Soil humus % was determined by colorimetric examination after extraction with 2.23% sodium pyrophosphate and 1% NaOH [33]. The total carbon (C) and nitrogen (N) were determined using an NC analyzer (SUMIGRAPH NCH-22F, Sumika Chemical Analysis Service, Osaka, Japan).

### Data analysis

The data were analyzed by three-way analysis of variance (ANOVA) using Crop-Statistical Tool for Agricultural Research, version 7.0 software (International Rice Research Institute, Philippines). Significant differences between means were compared using Fisher LSD test at p ≤ 0.05 using XLSTAT Version 2017 (Addinsoft). Principal component analysis (PCA) and correlation analysis were conducted with the yield and its related traits with different treatments using XLSTAT Version 2017 (Addinsoft).

## Results

### Nursery seedling growth response to TUAT-1 biofertilizer

Nursery application of TUAT-1 biofertilizer significantly increased shoot length in both Fukuhibiki and LTAT-29 genotypes (Table 1). There was no interaction effect of genotype and TUAT-1 biofertilizer on the growth of nursery seedlings. The growth-promoting effect of TUAT-1 biofertilizer was observed in the shoot and root fresh weight and shoot dry weight in both genotypes, although the increase was only significant for shoot fresh weight for both genotypes (Table 1). The density of TUAT-1 *Bacillus* cells in the fresh root of 21-day-old seedlings was 3.6 × 10^3^ and 4.2 × 10^3^ CFU g^−1^ fresh root in Fukuhibiki and LTAT-29, respectively.

#### Soil properties

The effect of biochar and biofertilizer, alone or combined, on soil physicochemical properties of post-harvest soil is shown in Table 2. The effect of BC was significant for CEC, total C (%), available K, and available Si. The interaction between biochar and biofertilizer was significant for total C (%) (S1 Table). Post-harvest soil analysis showed that NH_4_^+^-N, NO_3_^—^N, and total soil total N% did not differ significantly between treatments. The addition of biochar to the soil resulted in a significant increase in soil C content in both biochar treatments compared with the CT at the end of the experiment (Table 2). The biochar treatments with or without biofertilizer (BC and Bio+BC) also significantly increased the exchangeable K^+^ and slightly improved the exchangeable Mg^2+^ and available Si, however, no increase was observed for exchangeable Ca^+^ and available P. Soil humus % significantly increased with biochar treatments with or without biofertilizer (BC and Bio+BC). Soil EC and CEC measured at the end of the experiment were higher in BC than CT, although the differences were not statistically significant.

**Table 2:** Soil physicochemical properties after rice cultivation.

	Treatments			
Soil physicochemical properties	CT	Bio	BC	Bio+BC
pH (H_2_O)	5.80 ± 0.08^a^	5.50 ± 0.06^a^	5.40 ± 0.15^a^	5.50 ± 0.06^a^
EC (dS m^−1^)	0.44 ± 0.01^a^	0.58 ± 0.06^a^	0.62 ± 0.08^a^	0.51 ± 0.03^a^
CEC (cmol_c_ kg^−1^)	17.80 ± 0.42^a^	18.10 ± 0.28^a^	18.60 ± 0.35^a^	18.00 ± 0.42^a^
Total N (%)	0.26 ± 0.01^a^	0.26 ± 0.01^a^	0.25 ± 0.01^a^	0.25 ± 0.01^a^
Total C (%)	3.13 ± 0.15^c^	3.16 ± 0.11^c^	5.65 ± 0.14^a^	4.86 ± 0.08^b^
NH_4_ (mg kg^−1^)	31.00 ± 2.10^a^	25.90 ± 9.20^a^	28.90 ± 2.10^a^	22.00 ± 7.10^a^
NO_3_(mg kg^−1^)	1.00 ± 0.20^a^	2.00 ± 0.20^a^	1.00 ± 0.10^a^	1.00 ± 0.1^a^
Available P (mg kg^−1^)	90 ± 0.00^a^	100.50 ± 7.10^a^	90.50 ± 7.10^a^	100.00 ± 0.00^a^
Exchangeable K (mg kg^−1^)	320 ± 14.10^b^	335 ± 71.00^b^	420 ± 14.10^a^	450 ± 28.30^a^
Exchangeable Ca (mg kg^−1^)	3190 ± 21.2^a^	3070 ± 42.4^a^	3155 ± 7.10^a^	3055 ± 120.2^a^
Exchangeable Mg (mg kg^−1^)	700 ± 71.00^bc^	695 ± 71.00c	755 ± 21.20^ab^	775 ± 21.20^a^
Available Si (mg kg^−1^)	470 ± 21.2^a^	425 ± 21.2^a^	565 ± 77.8^a^	520 ± 56.6^a^
Humus %	5.40 ± 0.14^b^	5.65 ± 0.07^ab^	6.00 ± 0.07^a^	5.80 ± 0.14^a^

Mean ± SD followed by the same letter are not statistically significant using Fisher’s LSD test (p < 0.05).

### Growth

Neither biochar nor biofertilizer significantly affected plant height at different growth stages or tiller number at each WAT. However, the responses of the two genotypes for these traits showed significant differences between the treatments; in addition, there was a significant interaction effect of genotype × biochar. Although no interaction was observed between biochar×biofertilizer, the genotypic response of interaction (genotype×biochar×biofertilizer) was significant (Tables 3 and 4).

**Table 3:** Effect of TUAT-1 biofertilizer, biochar, and their combination treatments on plant height (cm) at different growth stages of two rice genotypes.

		Plant height (cm)			
		First tillering	Max. tillering	Booting	Heading
**Fukuhibiki**	**CT**	55.3 ± 1.9^e^	87.5 ± 2.8^d^	89.2 ± 2.1^c^	101.8 ± 1.0^d^
	**Bio**	60.7 ± 1.5^d^	88.8 ± 1.7^d^	92.8 ± 1.3^c^	102.3 ± 1.7^d^
	**BC**	57.7 ± 1.8^e^	90.8 ± 2.4^cd^	95.0 ± 3.7^c^	109.0 ± 5.4^c^
	**Bio+BC**	65.6 ± 1.4^c^	90.0 ± 3.6^cd^	93.0 ± 1.9^c^	104.5 ± 2.6^cd^
**LTAT-29**	**CT**	72.4 ± 0.8^ab^	94.2 ± 1.0^bc^	117.3 ± 7.6^b^	130.8 ± 4.3^b^
	**Bio**	72.9 ± 2.5^a^	96.0 ± 5.3^b^	109.3 ± 10.2^b^	129.0 ± 2.4^b^
	**BC**	70.1 ± 2.4^ab^	98.9 ± 3.8^ab^	129.5 ± 4.0^a^	140.0 ± 3.6^a^
	**Bio+BC**	69.6 ± 2.8^b^	102.0 ± 2.4^a^	126.0 ± 4.6^a^	137.0 ± 6.5^a^
**Analysis of variance**					
**Genotype (G)**		**	**	**	**
**BC**		ns	ns	ns	ns
**Bio**		ns	ns	ns	ns
**G**×**BC**		**	**	**	**
**G**×**Bio**		**	**	**	**
**BC**×**Bio**		ns	ns	ns	ns
**G**×**BC**×**Bio**		**	**	**	**

Mean ± SD followed by the same letter are not statistically significant using Fisher’s LSD test (p < 0.05). Note: Max. tillering: maximum tillering.

** p < 0.01

ns = not significant at 0.05 level

**Table 4:** Effect of TUAT-1 biofertilizer, biochar, and their combination treatments on tiller number at different growth stages of two rice genotypes.

		No. of tillers (WAT)				
		2	3	4	5	6
**Fukuhibiki**	**CT**	9 ± 0.5^b^	17 ± 1.7^ab^	24 ± 2.8^a^	25 ± 1.0^a^	25 ± 2.2^a^
	**Bio**	11 ± 1.5^a^	18 ± 1.9^a^	25 ± 0.5^a^	26 ± 0.8^a^	26 ± 1.0^a^
	**BC**	8 ± 2.1^b^	12 ± 2.3^c^	21 ± 3.8^b^	24 ± 4.1^a^	24 ± 2.9^a^
	**Bio+BC**	9 ± 0.5^b^	15 ± 1.5^b^	23 ± 1.3^ab^	24 ± 2.2^a^	24 ± 2.2^a^
**LTAT-29**	**CT**	6 ± 0.5^c^	12 ± 0.5^c^	14 ± 0.5^c^	14 ± 0.5^b^	14 ± 0.8^b^
	**Bio**	6 ± 0.8^c^	11 ± 1.3^cd^	15 ± 1.7^c^	15 ± 1.7^b^	15 ± 1.7^b^
	**BC**	6 ± 0.6^c^	9 ± 0.0^d^	13 ± 0.6^c^	14 ± 1.0^b^	14 ± 1.3^b^
	**Bio + BC**	6 ± 0.0^c^	9 ± 0.0^d^	13 ± 1.3^c^	13 ± 1.8^b^	13 ± 1.8^b^
**Analysis of variance**						
**Genotype (G)**		**	**	**	**	**
**BC**		ns	*	ns	ns	ns
**Bio**		ns	ns	ns	ns	ns
**G**×**BC**		**	**	**	**	**
**G**×**Bio**		**	**	**	**	**
**BC**× **Bio**		ns	ns	ns	ns	ns
**G**×**BC**×**Bio**		**	**	**	**	**

Mean ± SD followed by the same letter are not statistically significant using Fisher’s LSD test (p < 0.05).

* p < 0.05

** p < 0.01

ns = not significant at 0.05 level

At the first tillering (2 WAT) and maximum tillering (6 WAT) stages, the height of plants treated with only biochar (BC) did not significantly differ from those of plants treated with CT for both genotypes. However, biofertilizer with or without BC treatments (Bio and Bio+BC) of Fukuhibiki was significantly higher for first tillering, whereas plant height of LTAT-29 was significant higher at maximum tillering owing to Bio+BC than their respective controls (CTs). The plant height significantly increased with BC in both genotypes at the heading stage (Table 3). The increase in plant height with Bio+BC statistically increased in LTAT-29 and numerically increased in Fukuhibiki compared with their CTs. Generally, at the early tillering stage (2–4 WAT), tiller numbers after biochar treatments with or without biofertilizer (BC and Bio+BC) were significantly or numerically lower than those after CT. In each genotype, tiller number hill^−1^ showed no significant differences among the treatments at 5 and 6 WAT (Table 4).

### Physiological performance

The effect of biochar and biofertilizer, alone or combined, on the physiological properties of the rice plants are shown in Table 5. Biochar significantly affected SPAD at the tillering (p < 0.05) and heading stages (p < 0.05) and Pn at the heading stage (p < 0.01). The genotype was also significant for both SPAD and Pn, reflecting the different growth patterns between the genotypes. There were the interactions among genotype×biochar, genotype×biofertilizer, and genotype×biochar×biofertilizer but not between biochar and biofertilizer.

**Table 5:** Effect of TUAT-1 biofertilizer, biochar, and their combination treatments on chlorophyll content (SPAD) and net photosynthesis rate at different growth stages of two rice genotypes.

		SPAD			Pn		
		Tillering	Heading	Grain filling	Tillering	Heading	Grain filling
**Fukuhibiki**	**CT**	37.3 ± 2.0^b^	37.6 ± 0.9^bc^	37.7 ± 0.9^b^	10.5 ± 0.9^b^	11.0 ± 1.6^c^	11.9 ± 0.6^ab^
	**Bio**	38.3 ± 3.4^b^	37.9 ± 1.0^b^	37.6 ± 0.9^b^	10.4 ± 0.5^b^	11.7 ± 1.5^c^	11.3 ± 1.1^b^
	**BC**	39.8 ± 2.3^ab^	40.8 ± 0.8^a^	39.9 ± 0.6^a^	11.8 ± 2.0^ab^	12.4 ± 2.3^bc^	13.5 ± 1.7^a^
	**Bio+BC**	40.9 ± 3.2^ab^	40.2 ± 0.3^a^	39.0 ± 0.2^ab^	12.2 ± 0.9^ab^	13.2 ± 2.6^bc^	13.2 ± 1.6^a^
**LTAT-29**	**CT**	41.1 ± 2.3^ab^	35.3 ± 1.3^d^	31.9 ± 1.4^c^	11.8 ± 0.6^ab^	12.7 ± 1.2^bc^	8.4 ± 1.3^c^
	**Bio**	40.7 ± 1.0^ab^	36.2 ± 1.1^cd^	33.7 ± 1.6^c^	12.6 ± 1.2^a^	12.7 ± 0.3^bc^	8.4 ± 1.2^c^
	**BC**	42.0 ± 1.4^a^	38.2 ± 1.5^b^	32.4 ± 2.1^c^	12.5 ± 1.6^ab^	14.6 ± 0.1^ab^	9.1 ± 0.9^c^
	**Bio+BC**	42.4 ± 1.2^a^	37.8 ± 1.4^bc^	32.5 ± 2.2^c^	12.4 ± 1.3^ab^	15.8 ± 0.1^a^	9.0 ± 0.8^c^
**Analysis of variance**							
**Genotype (G)**		**	**	**	**	**	**
**BC**		*	**	ns	ns	**	ns
**Bio**		ns	ns	ns	ns	ns	ns
**G**×**BC**		**	**	**	*	**	**
**G**×**Bio**		*	**	**	ns	*	**
**BC**×**Bio**		ns	**	ns	ns	*	ns
**G**×**BC**×**Bio**		ns	**	**	ns	**	**

Mean ± SD followed by the same letter are not statistically significant using Fisher’s LSD test (p < 0.05).

* p < 0.05

** p < 0.01

ns = not significant at 0.05 level

The chlorophyll content (SPAD value) of plants that received biochar treatments with or without biofertilizer (BC and Bio+BC) was significantly higher than those that received CTs at the heading stage in both genotypes. Photosynthetic rate was numerically increased in LTAT-29 grown in biochar, and it significantly improved further when biochar and biofertilizer were used together at the heading stage, whereas in Fukuhibiki, there were no significant differences in the photosynthetic rate among the treatments, although at all stages, Pn was slightly higher for biochar treatments with or without biofertilizer (BC and Bio+BC) than for CT.

### Crop yield and biomass production

The effect of biochar and biofertilizer, alone or combined, on yield, yield components, and biomass production is shown in Table 6. The effect of the genotype was statistically significant for yield and all its components. The effect of biochar was significant (p < 0.05) for panicle weight, grain yield, and brown rice. The genotypic response to bioferilizer should be noted here, as shown by strong significant interactions between genotype×biofertilizer for almost all measured parameters. Genotypic interaction between biochar and biofertilizer (genotype×biochar×biofertilizer) was also significant.

**Table 6:** Effect of TUAT-1 biofertilizer, biochar, and their combination treatments on yield attributes of two rice genotypes.

		PN pot^−1^	PW	SB	UGB	GY	BR
			pot^−1^ (g)				
**Fukuhibiki**	**CT**	18 ± 1.5^a^	41.9 ± 2.1^a^	39.7 ± 1.2^f^	80.8 ± 5.7^d^	41.1 ± 2.1^a^	29.9 ± 1.7^ab^
	**Bio**	18 ± 1.7^a^	39.7 ± 1.8^ab^	42.6 ± 4.2^ef^	83.8 ± 2.7^d^	40.8 ± 1.9^ab^	30.2 ± 2.2^ab^
	**BC**	16 ± 1.5^b^	41.6 ± 0.8^a^	46.7 ± 2.9^de^	88.3 ± 2.4^d^	39.7 ± 0.8^a^	32.6 ± 0.9^a^
	**Bio+BC**	18 ± 1.7^a^	39.9 ± 3.0^ab^	49.5 ± 1.7^d^	89.4 ± 2.9^d^	39.1 ± 3.0^ab^	30.4 ± 3.4^ab^
**LTAT-29**	**CT**	12 ± 0.5^c^	30.6 ± 2.0^c^	73.9 ± 6.2^c^	104.5 ± 5.6^c^	28.7 ± 2.1^c^	19.5 ± 1.7^d^
	**Bio**	12 ± 1.0^c^	28.2 ± 2.8^c^	82.6 ± 4.2^ab^	110.8 ± 1.8^bc^	26.3 ± 2.3^c^	17.3 ± 2.3^d^
	**BC**	10 ± 0.6^d^	41.2 ± 1.5^a^	78.1 ± 2.9^bc^	119.3 ± 2.6^ab^	37.1 ± 5.5^ab^	28.1 ± 1.6^b^
	**Bio+BC**	11 ± 0.6^cd^	36.9 ± 3.6^b^	85.8 ± 4.8^a^	122.6 ± 3.3^a^	35.6 ± 3.6^ab^	23.3 ± 3.5^c^
**Analysis of variance**							
**Genotype (G)**		**	**	**	**	**	**
**BC**		ns	*	ns	ns	*	*
**Bio**		ns	ns	ns	ns	ns	ns
**G**×**BC**		**	**	**	**	**	**
**G**×**Bio**		**	**	**	**	**	**
**BC**×**Bio**		ns	*	ns	ns	ns	ns
**G**×**BC**×**Bio**		**	**	**	**	**	**

Mean ± SD followed by the same letter are not statistically significant using Fisher’s LSD test (p < 0.05). Note: PN = Panicle number, PW = Panicle weight, SB = Straw biomass, UGB = Upper-ground biomass, GY = Grain yield, BR = Brown rice.

* p < 0.05

** p < 0.01

ns = not significant at 0.05 level

The highest grain yield was observed for Fukuhibiki than that for LTAT-29. The grain yields after the CT, Bio, BC, and Bio+BC were 41.1, 40.8, 39.7, and 39.1 g pot^−1^, respectively, in Fukuhibiki, whereas those after the CT, Bio, BC, and Bio+BC were 28.7, 26.3, 37.1, and 35.6 g pot^−1^, respectively, in LTAT-29. With respect to Fukuhibiki, there were no significant differences in the grain yield between the treatments. Meanwhile, compared with CT, biochar treatments with or without biofertilizer (BC and Bio+BC) significantly increased the grain yield in LTAT-29. A similar trend was observed for brown rice.

Generally, higher panicle and panicle weight were observed in Fukuhibiki than those in LTAT-29. BC decreased the panicle number in Fukuhibiki; however, no significant differences in the panicle weight were observed between treatments of BC and CT. Meanwhile, LTAT-29 treated with biochar with or without biofertilizer (BC and Bio+BC) showed moderate decrements in panicle number, although panicle weights were significantly higher than those shown by CT and Bio (Table 6), with significant increases of 35% for BC and 30% for Bio+BC compared with CT.

Straw biomass production in LTAT-29 was significantly higher than that of Fukuhibiki. In Fukuhibiki, biochar treatments with or without biofertilizer (BC and Bio+BC) significantly increased straw biomass (by 18% and 25%, respectively) compared with CT. Meanwhile, in LTAT-29, Bio with or without BC (Bio and Bio+BC) significantly enhanced straw biomass compared with CT. Although LTAT-29 treated with BC showed numerically increased straw biomass, but it did not reach a significant level. Similarly, LTAT-29 showed a higher above-ground biomass production than Fukuhibiki. Treatment effects on above-ground biomass production were not observed for Fukuhibiki; however, above-ground biomass production was significantly increased by biochar treatments with or without biofertilizer (BC and Bio+BC) in LTAT-29.

### Nutrient uptake in straw and grain

Biochar and biofertilizer, alone or in combination, significantly affected plant nutrient concentration, but the effect largely depended on the rice genotype (Table 7). Straw N content was significantly increased by Bio in LTAT-29, followed by Bio+BC compared with CT. However, in Fukuhibiki, a significant increase in straw N content was only observed in Bio+BC compared with CT. Overall, the combination of BC and Bio had a positive effect on N uptake in both genotypes. There was no significant difference in grain N uptake between the treatments in Fukuhibiki; however, grain N uptake was significantly increased by BC; grain N uptake was slightly increased by Bio+BC in LTAT-29. BC and Bio differentially affected straw P uptake in the two rice genotypes. In Fukuhibiki, the greatest P uptake was observed in Bio+BC, followed by Bio and then BC, with CT showing the lowest P uptake. In contrast, P uptake in LTAT-29 was highest with CT, lowest with BC, with Bio and Bio+BC in between. Straw K uptake was strongly influenced by BC, Bio, and their combination in the two rice genotypes. In Fukuhibiki, Bio+BC showed the highest straw K uptake, followed by BC and Bio, with CT the lowest. However, in LTAT-29, the lowest K uptake was observed with Bio, whereas slightly increased straw K uptake was observed with BC and significant increase with Bio+BC. This indicated that the influence of Bio on K uptake depends on the rice genotype.

**Table 7:** N, P, and K uptake (mg plant^−1^) in two forage rice in response to different treatments.

		N		P		K	
		straw	grain	straw	grain	straw	grain
**Fukuhibiki**	**CT**	223.7 ± 31.8^d^	488.1 ± 44.2^a^	66.1 ± 9.0^e^	129.0 ± 9.0^a^	786.1 ± 42.9^f^	151.9 ± 11.2^a^
	**Bio**	266.8 ± 11.8^cd^	494.7 ± 10.0^a^	93.8 ± 6.6^d^	131.7 ± 5.2^a^	936.3 ± 60.5^e^	145.0 ± 8.0^a^
	**BC**	276.2 ± 40.1^cd^	482.6 ± 44.3^a^	83.6 ± 10.8^d^	134.9 ± 7.2^a^	1120.1 ± 65.2^d^	154.6 ± 12.2^a^
	**Bio+BC**	303.2 ± 16.9^c^	476. 7 ± 35.6^a^	107.8 ± 7.1^c^	132.9 ± 7.1^a^	1205.1 ± 24.3^c^	141.7 ± 11.0^a^
**LTAT-29**	**CT**	371.6 ± 34.7^b^	289.5 ± 20.0^cd^	130.7 ± 14.5^a^	91.7 ± 6.9^bc^	1264.0 ± 68.6^bc^	81.2 ± 5.3^bc^
	**Bio**	427.2 ± 22.4^a^	267.5 ± 65.6^d^	121.9 ± 7.0^ab^	81.2 ± 18.4^c^	1139.2 ± 20.0^d^	71.8 ± 15.6^c^
	**BC**	370.1 ± 11.7^b^	356.9 ± 17.3^b^	110.0 ± 7.4^bc^	103.6 ± 10.0^b^	1275.4 ± 12.2^ab^	96.1 ± 8.0^b^
	**Bio+BC**	405.4 ± 22.4^ab^	332.9 ± 34.2^bc^	122.8 ± 7.5^ab^	104.0 ± 9.1^b^	1337.1 ± 41.4^a^	95.8 ± 10.3^b^
**Genotype (G)**		**	**	**	**	**	**
**BC**		ns	ns	ns	ns	**	ns
**Bio**		ns	ns	ns	ns	ns	ns
**G**×**BC**		**	**	**	**	**	**
**G**×**Bio**		**	**	**	**	**	**
**BC**×**Bio**		ns	ns	ns	ns	**	ns
**G**×**BC**×**Bio**		**	**	**	**	**	**

Mean ± SD followed by the same letter are not statistically significant using Fisher’s LSD test (p < 0.05).

** p < 0.01

ns = not significant at 0.05 level

**Fig 1:**
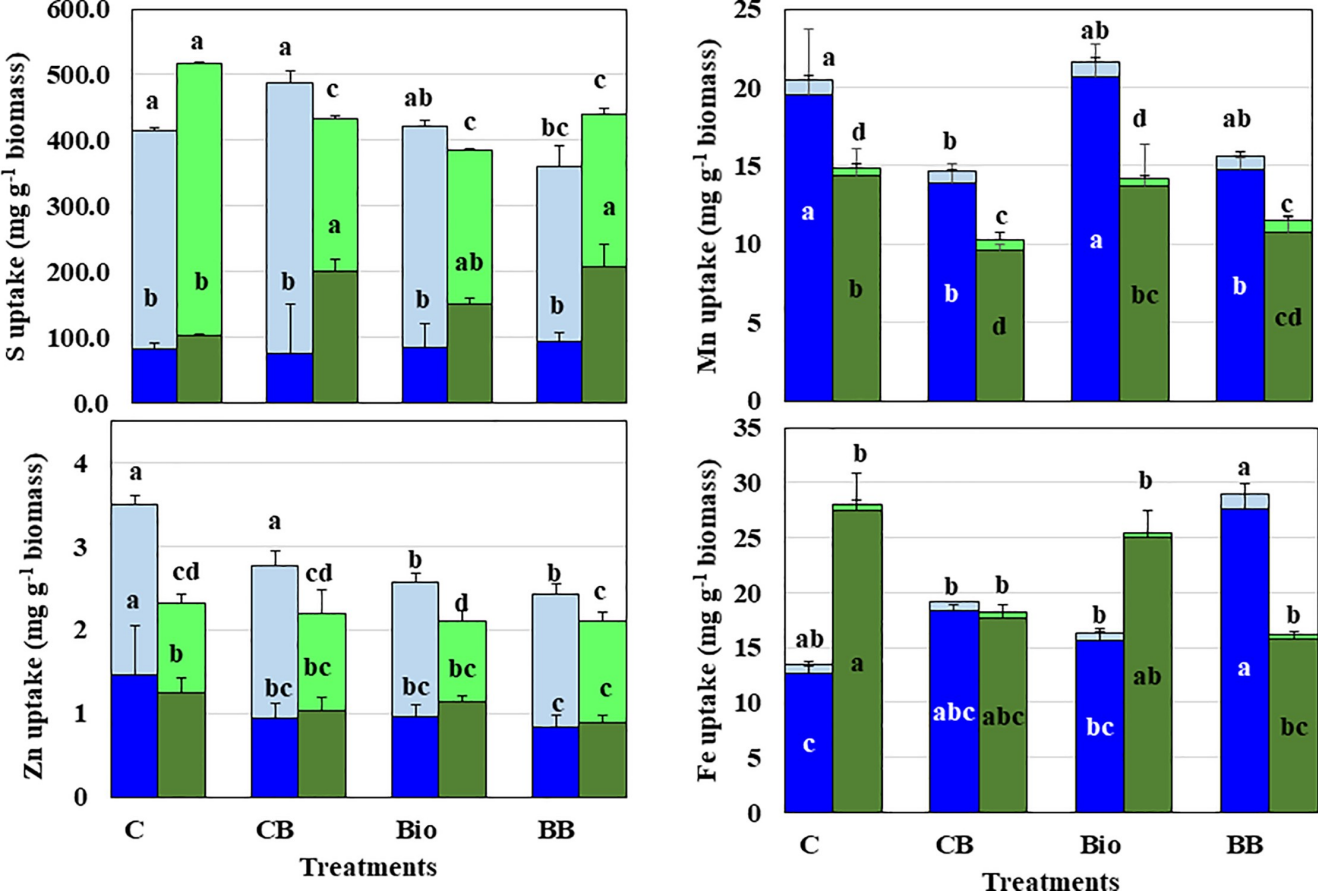
S, Zn, Mn, and Fe uptake in two forage rice genotypes in response to biochar, TUAT-1 biofertilizer and their combination. Blue and light blue bars represent straw and grain uptake in Fukuhibiki, respectively, and green and light green bars represent straw and grain uptake in LTAT-29, respectively. Data are provided as mean ± SD. Values indicated by the same letter are not significantly different at the 5% level by Fisher’s LSD test (p < 0.05).

The trends in S, Zn, Mn, and Fe uptake in response to treatments were also different between the two genotypes (Fig 1 and S2 Table). In Fukuhibiki, there was no significant difference in straw S uptake among the treatments; however, in LTAT-29, treatments with biochar alone or in combination with biofertilizer (BC and Bio+BC) significantly increased straw S uptake. Grain S uptake significantly decreased with to Bio+BC in Fukuhibiki, whereas grain S uptake significantly decreased with Bio, BC or Bio+BC in LTAT-29 compared with their respective CTs.

BC application strongly influenced the micronutrient uptake (Mn, Zn, and Fe) in both rice genotypes. In both genotypes, treatments with biochar with or without biofertilizer (BC and Bio+BC) significantly decreased Mn uptake in straw compared with their respective CTs. Grain Mn uptake was significantly decreased with BC in Fukuhibiki, whereas the lowest grain Mn uptake was observed with Bio in LTAT-29. Straw Zn uptake was significantly lower in Bio, BC, and Bio+BC compared with CT in Fukuhibiki. In contrast, neither Bio nor BC significantly decreased straw and grain Zn uptake compared with CTs, but Bio+BC significantly decreased straw Zn uptake in LTAT-29. Meanwhile, in Fukuhibiki, the treatments of biofertilizer alone or in a combination with biochar (Bio and Bio+BC) showed a significant decrease in grain Zn uptake compared with CT. Generally, we observed that treatment with either BC or Bio decreased Zn uptake in both genotypes.

Grain Fe uptake was unchanged among the treatments in LTAT-29; Bio+BC resulted in significantly lower Fe uptake in straw than CT. In Fukuhibiki, no change was observed in straw and grain Fe uptake; however, Bio+BC significantly increased straw Fe uptake.

### Nitrogen use, utilization, and uptake efficiencies

**Fig 2:**
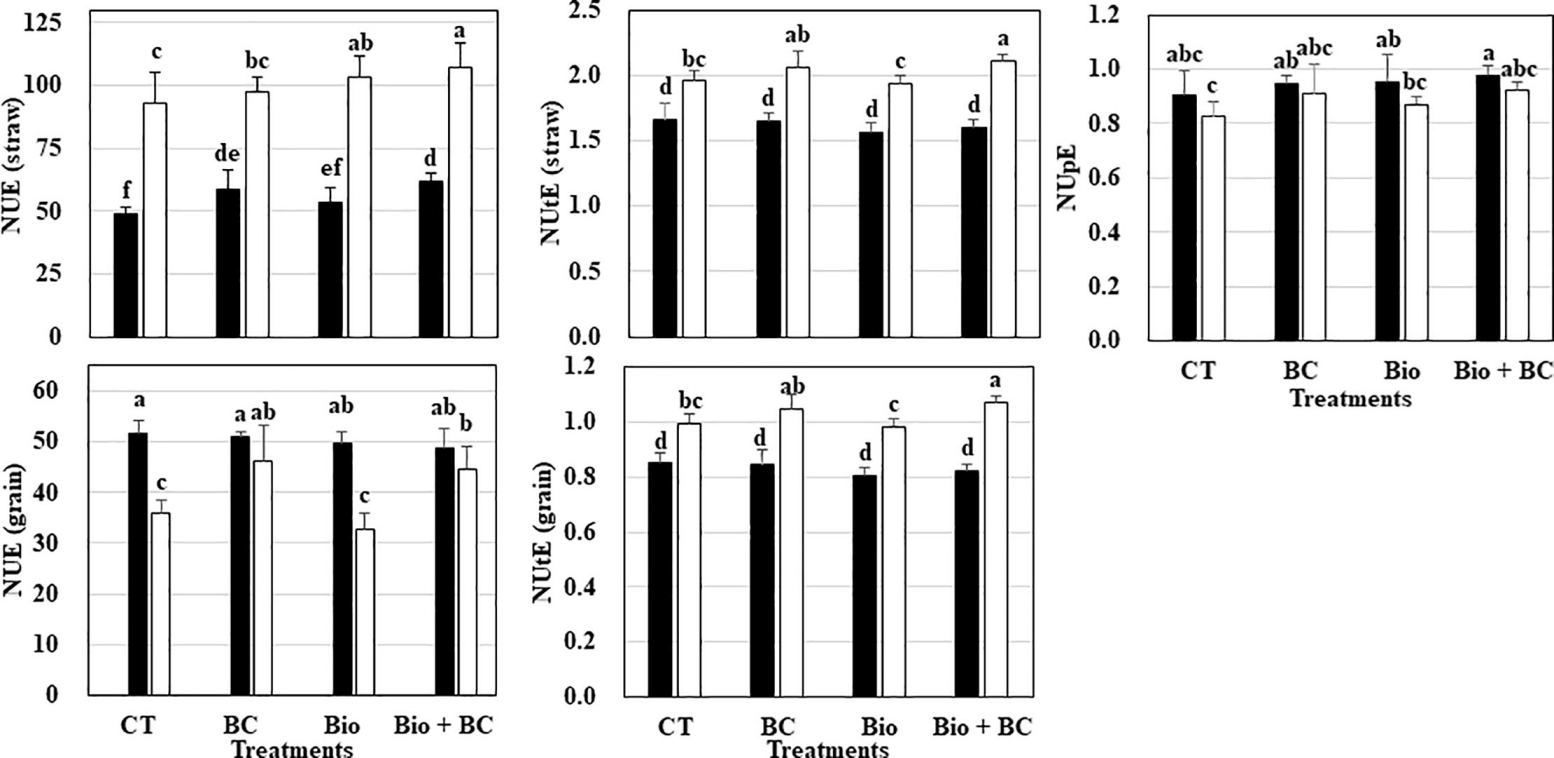
Nitrogen Use Efficiency parameters of two forage rice genotypes in response to different treatments. Black and white bars represent Fukuhibiki and LTAT-29. Data are given as mean ± SD. Values indicated by the same letter are not significantly different at the 5% level by Fisher’s LSD test (p < 0.05).

Generally either NUE or NUtE in straw of LTAT-29 is significantly higher than that of Fukuhibiki. Treatment with biochar or biofertilizer, or their combination, influenced NUE and NUtE in straw and grain depending on the rice genotype (Fig 2). In Fukuhibiki, BC and Bio+BC significantly increased NUE. Bio slightly improved NUE, but the influence was not significant. In LTAT-29, although BC slightly increased NUE, Bio and Bio+BC significantly increased NUE in straw. NUE in grain of LTAT-29 was significantly increased with BC and Bio+BC. Neither BC nor Bio alone or in combination influenced NUtE in straw or grain in Fukuhibiki; however, BC slightly improved these two factors, and a significant increase was found with Bio+BC in LTAT-29. Interestingly, NUpE, representing the capture of (unlimited) N in solution from the soil, paralleled the increase in the biomass. TUAT-1 biofertilizer alone or in a combination with biochar slightly increased NUpE in both genotypes, but the increase was not statistically significant.

### Principal component analysis

**Fig 3:**
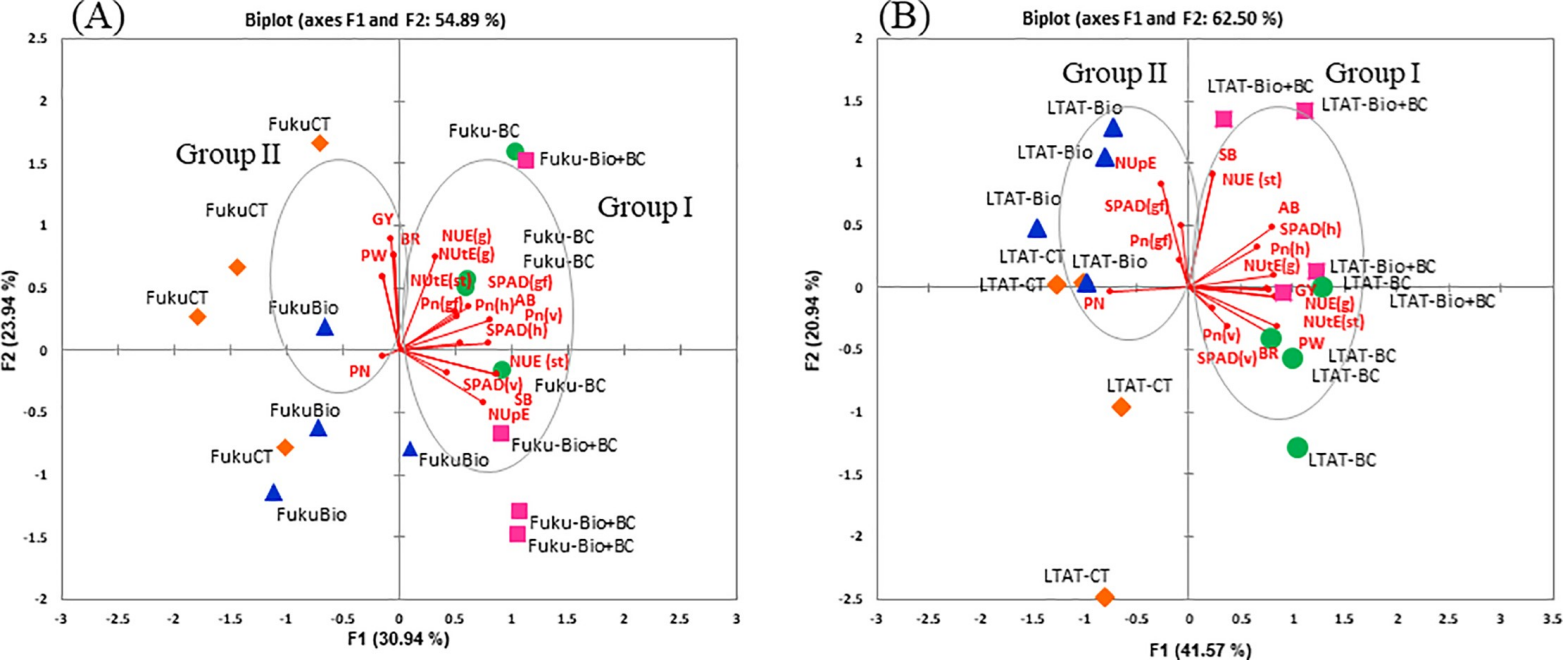
Principal component analysis (PCA) of yield and its related traits of different treatments (A) for Fukuhibiki and (B) for LTAT-29. (n = 4). Note: PN: panicle number, PW: panicle weight, SB: straw biomass, AB: above-ground biomass: GY: grain yield, BR: brown rice, NUE (g) or NUE (st): NUE in grain and straw, NUtE (g) or NUtE (st): NUtE in grain and straw, SPAD and P (v) (h) (gf): SPAD and Pn at vegetative, heading and grain filling stage, respectively. Rhombus filled with orange, triangle filled with blue, circle filled with green and square filled with pink represent the treatments of CT (control), Bio (biofertilizer), BC (biochar) and biofertilizer + biochar, respectively.

PCA revealed patterns in plant parameters across all samples (Fig 3A and 3B). In LTAT-29, PCA of above-ground biomass, SPAD at heading, Pn at heading, NUtE grain, grain yield, NUtE straw, NUE grain, brown rice, panicle weight, SPAD at vegetative and Pn at vegetative were mostly influenced by BC and Bio+BC (group I). NUpE, SPAD at grain filling, Pn at grain filling, and panicle number (group II) were influenced by CT and Bio (Fig 3B). Grain yield was highly associated with NUtE in grain and straw, NUE in grain and Pn at heading and negative relationship with panicle number (S5 Table). However, the opposite was true for Fukuhibiki (Fig 3A). The samples showed slight scattering from groups of parameters. Grain yield, brown rice, panicle weight, and panicle number were grouped together and were mostly associated with CT.

## Discussion

In the present study, both genotypes showed positive effects on biomass production following biochar application (BC). The impact of biochar on crop yield is very complex. To date, the effects of biochar application on crop yield are controversial and highly variable; for example, biochar has been reported to increase crop yields, including those of rice [14]; however, Rajkovich et al. [34] reported that the effect of biochar on crop yields was negative. These highly diverse results are not surprising because various effects of biochar application on a cropping yield have mainly been ascribed to the characteristics and rate of the biochar used, soil type, crop species, climate condition, and other factors. In our study, we have clearly pointed out that the effect of biochar on forage rice yield was depended on the genotype; specifically, a yield increment owing to biochar was only observed in LTAT-29 but not in Fukuhibiki (Table 6). A recent study of the influence of biochar on different growth traits in tomato showed differential effects depending on the tomato variety, suggesting that the effect of biochar on crop growth promotion is dependent on genotype [35], and that the gibberellin pathway may play a role in biochar-mediated growth promotion.

Beneficial physiological outcomes on rice yield using biochar were only observed in LTAT-29. Improved photosynthesis at the heading stage might have given rise to the greater seed filling in LTAT-29 since we observed that a decrease in panicle number in LTAT-29 did not result in a decline in panicle weight or seed grain weight following biochar treatments (Tables 5 and 6). Similarly, cultivars with large or extra-heavy panicles frequently do not fulfill their high yield potential owing to poor grain filling or slow grain filling rate [36, 37]. It is well reported that > 70% of grain yield comes from assimilates produced in the post-anthesis period, and a high photosynthetic rate during heading may be desirable to sustain source activity [38]. On confirmation via PCA and correlation analysis, it was found that BC highly affected Pn at the heading stage, and this is significantly correlated with grain yield (Fig 3B and S5 Table).

However, this was not the case for Fukuhibiki (Fig 3A and S4 Table). Although in Fukuhibiki, treatments with biochar alone or in a combination with biofertilizer led to increase NPK uptake, SPAD value, and Pn rate at heading, which were evident via enhancement in plant size (plant biomass), but did not lead to increase in grain yield (Table 6). It is reasonable to consider that different genotypes may vary with respect to nutrient uptake, accumulation, partitioning, and remobilization in rice plants during growth. Here, in our study, we postulated that enhancing net photosynthesis with biochar may result in better sink capacity and fertilized seed per panicle and finally increase the grain yield in large panicle type LTAT-29.

Biochar is effective in changing the physical, chemical, and biological properties of soil [6–8], thereby increasing growth and yield of crop. We observed that the addition of BC significantly increased total C and the C/N ratio in the rhizosphere, and these findings were consistent with those in other reports in the literature [14, 39]. Surprisingly, although the same rate of biochar (2%) was applied, TC content was significantly lower in BC+Bio than in BC (Table 2). Similar findings were reported by Yusif *et al*. [40], who found that rhizobium inoculation decreased the soil pH and organic carbon content. They suggested that the decrease in organic carbon was attributed to increased microorganisms that hasted the decomposition of organic carbon in the rhizosphere. In our study, we postulated that changes in root exudates or rhizodeposition by rice plants owing to TUAT-1 inoculation may be explained [41, 42], thereby attracting and selecting microorganisms in the rhizosphere. Microorganisms can alter the properties of biochar, especially when causing biochar to oxidize the surface of particles, which increases the oxygen content and decreases the carbon content in biochar particles [43].

In line with the results reported by Yang *et al*. [44], we found that N content in soils amended by rice-husk biochar in the current study was not elevated relative to that of the control. In addition, our results did not show any enhancement of other soil chemical properties, such as pH, EC, CEC, NH_4_^+^, NO_3_^-^, exchangeable Mg and Ca, and available P. However, BC application improved the exchangeable K content of soil. Mengel and Kirkby [45] reported that a high K content in BC contributed to more plant-available K in the soil. Soil humus % was raised following BC treatment with or without biofertilizer. Similarly, a short-term laboratory incubation experiment showed that biochar incorporated into soil plays a positive role in promoting the formation of soil humus [46–47], which may be beneficial to the stability of soil aggregate stability. Soil chemical properties, such as available P, EC, NO_3_^−^, and humus %, slightly improved with biofertilizer application, although not statistically significantly, suggesting that the use of TUAT-1 inoculant biofertilizer did not influence soil physicochemical properties.

In our study, genotype responses to both BC and Bio were significant, with differences in the nutrient uptake (Table 7). Grain N uptake was increased with BC, whereas Bio increased straw N uptake in LTAT-29. We suggest that significantly improved straw N uptake due to Bio in LTAT-29 results in an increase in straw biomass. Reflecting the findings of Win *et al* [21] and Torii [20], growth promotion by inoculation with biofertilizer was accompanied by increased N uptake in all plant tissues resulting in the promotion of biomass in forage rice. This suggests either an increase in searching for nutrients or the stimulation of direct uptake of water and nutrients in field conditions, leading to improved whole-plant growth. In Fukuhibiki, in contrast, N uptake remained unchanged with either biofertilizer or BC. However, the interaction between genotype×biofertilizer×biochar was significant. Straw N uptake was slightly improved with either BC or Bio; however, their combination (Bio+BC) resulted in a significant straw N uptake. It is suggested that the Bio+BC combination had a positive effect on tissue N uptake resulting in the significant increase in straw and above-ground biomass production.

There was a significant trend of increased K accumulation in straw in Fukuhibiki in the order Bio+BC > BC > Bio > CT (Table 7). Several *Bacillus* spp. have been reported for their ability to enhance the solubilization and uptake of K [48, 49] and P [50]. A similar trend was observed for P uptake. Increased nutrient uptake by plants inoculated with PGPB has been attributed to the production of plant growth regulators at the root interface, which stimulates root development and results in better absorption of water and nutrients from the soil [51]. The capacity of TUAT-1 to influence plant growth, water, and nutrient content has been widely reported [20–22]. In contrast, BC decreased P uptake in straw and plant tissue and Bio decreased K uptake in LTAT-29.

Our study indicates that different rice genotypes exhibit different strategies regarding macro-nutrient uptake with these treatments, i.e., a genotype dependency. A positive effect of incorporation of biochar and biofertilizer on nutrient uptake (N and K) was observed for both genotypes. The increase in nutrient uptake is attributed to the release of some nutrients (particularly K) by biochars [52] and help in preventing fertilizer run off, leaching, and retaining moisture [11, 12], and promotion of root growth by altering root architecture due to TUAT-1 strain [21] may lead to increase in nutrient uptake for both genotypes.

Biochar application also significantly affected plant uptake of Mn, Fe, and Zn, with variations depending on the genotype (Fig 1 and S2 Table). A significant decrease in straw Mn uptake was observed with BC (BC, Bio+BC) for both genotypes, whereas a significant decrease in straw Zn uptake with Bio or BC was only observed for Fukuhibiki. Contrasting results were observed for the two genotypes with respect to Fe, whereby a significant increase in Fe uptake was observed with Bio+BC in Fukuhibiki but a decrease was observed for LTAT-29 (Fig 1). Although the mechanism of genotype response in Fe uptake with different treatments remains unclear, we found similar trends of P concentration in tissues among the treatments for the two genotypes (Table 7). Marschner *et al*. [53] reported that iron mobilization strategies of plants and microorganisms involve exudation of organic anions with Fe-complexation abilities. This additionally contributes to increasing P availability to plants through desorption from oxides or dissolution of precipitated phosphates, suggesting that P uptake by plants is related to Fe uptake by plants. Changes in the release of exudates from the roots of two genotypes and soil microbes under different treatments may affect the solubility of P and Fe, which can differ the uptake of these ions in plant tissues.

Together, these results demonstrate that plant genotype influences nutrient uptake activity following treatment with either Bio or BC. So, in our study, it is not surprising that the two forage rice genotypes differed in their capacity for soil nutrient uptake characteristics under the same treatment conditions. Factors to be considered include differences in the contact surface area between roots and soil [54], in the composition and amount of root exudates [55], and in the rhizosphere microflora [56], all of which may result in differences in the chemistry and biology of the rhizosphere. In addition, the availability of nutrients in the rhizosphere is controlled by the combined effects of soil properties, plant characteristics, and the interaction of roots with microorganisms [55]. These factors might be involved in the differential effects of Bio, BC, or a combination of Bio and BC on two different genotypes.

Plant growth is related to nutrient uptake from the growth medium and improvement of nitrogen utilization efficiency. Improving the efficiency of nitrogen (N) uptake and utilization in plants could increase crop yields, while reducing N fertilization and subsequently, environmental pollution [57]. In our study, NUE of straw in both genotypes increased (significantly or numerically) with either BC or Bio, and the highest NUE was observed with the combination, Bio+BC (Fig 2). There are reports showing that BC has the potential to reduce N leaching [58, 59] and adsorb NH_4_ [60, 61] in agricultural soils, implying that the higher fertilizer N uptake under BC was associated with a decreased fertilizer N loss.

Although not tested in the present work, we suggest that a significant improvement of NUE and the highest values for total N uptake in both genotypes with Bio+BC (Fig 2 and Table 7) are related to the role played by BC in reducing N losses [11–12] and *Bacillus pumilus* TUAT-1 strain in altering root architecture and growth owing to the release of phytostimulators [62]. In addition, we observed that Bio+BC increased NUtE in straw and grain in LTAT-29. It is suggested that N take up is effectively used for and reflects better straw and grain yield production (Table 6). However, a similar trend was not observed in Fukuhibiki. Therefore, in this study, it is noted that the positive effects of Bio and BC on N efficiency are genotype-dependent in rice (S3 Table). It was clearly described in PCA that BC in LTAT-29 has an impact on a group of the traits: NUtE in straw and grain and NUE in grain and Pn and SPAD at heading affected grain yield positively (Fig 3B and S5 Table), whereas Bio had a high impact on straw biomass, NUE in straw, and above-ground biomass. However, these positive effects of either biochar or biofertilizer on grain yield and relative traits were not observed for Fukuhibiki (Fig 3A).

Taken together, application of biochar shows promise as an ecologically sound technology for improvement of soil quality and nutrient availability as well as crop productivity in forage rice. The results of our study indicate that biochar or biofertilizer shows a variable impact on plant growth depending on the two forage genotypes (Fig 3). The synergistic effect of biochar and biofertilizer is considered to be the result of an increased plant nutrient uptake leading to enhancement in biomass production and some physiological performance for both genotypes.

## Conclusion

Our study has revealed that both biochar and biofertilizer can alter plant growth and nutrient uptake in two forage rice genotypes. In general, amendment with biochar had a greater effect than biofertilizer on plant growth and soil physicochemical properties. Application of rice-husk biochar, with the utilization of local resources, shows potential as an environmentally friendly technology for improvement of soil physicochemical properties and crop productivity in forage rice genotypes. Overall, the positive effects of biochar were observed on plant physiology, biomass production, and grain yield. However, grain yield and nutrient uptake differed in response to biochar application, possibly dependent on plant traits and plant genetic composition. In addition, the effect of biofertilizer on growth, yield, and nutrient uptake also depends on rice genotype; thus, the effect on growth promotion and yield with biofertilizer in different rice genotypes under field conditions warrants further investigation.

These results are partly supported by the hypothesis that biochar incorporation into the soil influences interactions between plant genotypes and biofertilizer that are linked with plant nutrient uptake. A positive combined effect of biochar and biofertilizer was observed for nutrient uptake, although it has a genotypic dependency, so it can be concluded that both biochar and biofertilizer have the potential to improve nutrient supply and uptake and can thus be used for sustainable agriculture.

Overall, genotype-dependent differences in response to biochar highlight the need to consider plant genetic composition as well as production characteristics of the biochar (source biomaterials, carbonization conditions), application rates, and soil type, topics worthy of future study.

## Supporting information

S1 Table Results of the three-way ANOVA analysis for the effect of biochar and TUAT-1 biofertilizer and their combinations on post-harvest soil properties. (DOCX)

S2 Table Results of the three-way ANOVA analysis of F value for the effect of biochar and TUAT-1 biofertilizer and their combinations in two genotypes on S, Mn, Zn and Fe uptake (mg plant-1). (DOCX)

S3 Table Results of the three-way ANOVA analysis of F value for the effect of biochar and TUAT-1 biofertilizer and their combinations in two genotypes on N use efficiency. (DOCX)

S4 Table Correlation matrix (Pearson (n) for grain yield and its related traits for different treatments of Fukuhibiki. (DOCX)

S5 Table Correlation matrix (Pearson (n) for grain yield and its related traits for different treatments of LTAT-29. (DOCX)
