# Supplementary material for: Influence of rice-husk biochar and Bacillus pumilus strain TUAT-1 on yield, biomass production, and nutrient uptake in two forage rice genotypes
Source: PLoS One. 2019 Jul 31;14(7):e0220236. doi: 10.1371/journal.pone.0220236 (PMC6668810; doi:10.1371/journal.pone.0220236)
Supplement: S1 Table — (DOCX) [file pone.0220236.s001.docx]

S1 Table. Results of the three-way ANOVA analysis for the effect of biochar and TUAT-1 biofertilizer and their combinations on post-harvest soil properties

| Analysis of variance | BC | Bio | Bio × BC |
| --- | --- | --- | --- |
| pH (H_2_O) | ns | ns | ns |
| EC (dS m^−1^) | ns | ns | ns |
| CEC (cmol_c_ kg^−1^) | * | ns | ns |
| Total N (%) | ns | ns | ns |
| Total C (%) | ** | ns | ** |
| NH_4_ (mg kg^−1^) | ns | ns | ns |
| NO_3_(mg kg^−1^) | ns | ns | ns |
| Available P (mg kg^−1^) | ns | ns | ns |
| Exchangeable K (mg kg^−1^) | * | ns | ns |
| Exchangeable Ca (mg kg^−1^) | ns | ns | ns |
| Exchangeable Mg (mg kg^−1^) | ns | ns | ns |
| Available Si (mg kg^−1^) | * | ns | ns |
| Humus % | ns | ns | ns |

* p < 0.05, ** p < 0.01, ns = not significant at 0.05 level
