# Supplementary material for: Influence of rice-husk biochar and Bacillus pumilus strain TUAT-1 on yield, biomass production, and nutrient uptake in two forage rice genotypes
Source: PLoS One. 2019 Jul 31;14(7):e0220236. doi: 10.1371/journal.pone.0220236 (PMC6668810; doi:10.1371/journal.pone.0220236)
Supplement: S2 Table — (DOCX) [file pone.0220236.s002.docx]

S2 Table. Results of the three-way ANOVA analysis of F value for the effect of biochar and TUAT-1 biofertilizer and their combinations in two genotypes on S, Mn, Zn and Fe uptake (mg plant^-1^)

|  | S | | Mn | | Zn | | Fe | |
| --- | --- | --- | --- | --- | --- | --- | --- | --- |
|  | straw | grain | straw | grain | straw | grain | straw | grain |
| Genotype (G) | 16.3 **(0.002)** | 4.5 (0.056) | 52.9 **(0.000)** | 133.5 **(0.000)** | 0.1 (0.744) | 81.9 **(0.000)** | 1.2 (0.292) | 8.8 **(0.012)** |
| BC | 8.7 **(0.026)** | 6.8 **(0.040)** | 20.7 **(0.004)** | 1.4 (0.286) | 8.6 **(0.026)** | 0.6 (0.257) | 0.03 (0.868) | 0.5 (0.494) |
| Bio | 0.6 (0.480) | 9.5 **(0.022)** | 2.6 (0.155) | 0.2 (0.692) | 5.3 (0.062) | 44.6 **(0.001)** | 0.6 (0.484) | 0.1 (0.799) |
| BC × Bio | 0.1 (0.819) | 0.1 (0.780) | 1.1 (0.334) | 1.1 (0.326) | 1.0 (0.354) | 10.5 **(0.018)** | 0.4 (0.553) | 3.7 (0.103) |
| G × BC | 3.5 (0.087) | 2.9 (0.115) | 2.0 (0.178) | 16.6 **(0.002)** | 0.3 (0.615) | 3.9 (0.073) | 12.5 **(0.004)** | 0.7 (0.408) |
| G × Bio | 0.12 (0.675) | 0.1 (0.724) | 0.3 (0.622) | 2.1 (0.177) | 1.3 (0.275) | 4.9 **(0.048)** | 2.5 (0.138) | 2.2 (0.162) |
| G × BC × Bio | 0.4 (0.518) | 8.6 **(0.013)** | 0.6 (0.463) | 0.00 (0.969) | 2.2 (0.161) | 0.0 (0.939) | 0.3 (0.589) | 0.8 (0.401) |

p-values are shown in brackets. Values in bold indicate statistically significant differences (p < 0.05).
