# Supplementary material for: Influence of rice-husk biochar and Bacillus pumilus strain TUAT-1 on yield, biomass production, and nutrient uptake in two forage rice genotypes
Source: PLoS One. 2019 Jul 31;14(7):e0220236. doi: 10.1371/journal.pone.0220236 (PMC6668810; doi:10.1371/journal.pone.0220236)
Supplement: S3 Table — (DOCX) [file pone.0220236.s003.docx]

S3 Table. Results of the three-way ANOVA analysis of F value for the effect of biochar and TUAT-1 biofertilizer and their combinations in two genotypes on N use efficiency

|  | NUE | | NUtE | | NUpE |
| --- | --- | --- | --- | --- | --- |
|  | straw | grain | straw | grain |  |
| Genotype (G) | 559.8  **(0.000)** | 57.6  **(0.000)** | 231.4  **(0.000)** | 219.7  **(0.000)** | 164.9  **(0.000)** |
| BC | 16.2  **(0.004)** | 13.7  **(0.034)** | 45.4  **(0.007)** | 32.1  **(0.011)** | 5.37  (0.103) |
| Bio | 13.5  **(0.011)** | 2.6  (0.156) | 0.62  (0.462) | 0.65  (0.450) | 48.8  **(0.0004)** |
| BC × Bio | 0.04  (0.857) | 0.04  (0.845) | 0.6  (0.481) | 2.3  (0.153) | 1.49  (0.268) |
| G × BC | 1.48  (0.247) | 18.3  **(0.001)** | 6.65  (0.024) | 5.8  **(0.0032)** | 7.1  **(0.021)** |
| G × Bio | 2.62  (0.132) | 0.03  (0.863) | 2.58  (0.134) | 2.05  (0.1774) | 0.64  (0.439) |
| G × BC × Bio | 0.00  (0.972) | 0.06  (0.810) | 0.10  (0.744) | 0.1  (0.761) | 0.13  (0.720) |

p-values are shown in brackets. Values in bold indicate statistically significant differences (p < 0.05).
