# Supplementary material for: Influence of rice-husk biochar and Bacillus pumilus strain TUAT-1 on yield, biomass production, and nutrient uptake in two forage rice genotypes
Source: PLoS One. 2019 Jul 31;14(7):e0220236. doi: 10.1371/journal.pone.0220236 (PMC6668810; doi:10.1371/journal.pone.0220236)
Supplement: S4 Table — (DOCX) [file pone.0220236.s004.docx]

S4 Table. Correlation matrix (Pearson (n) for grain yield and its related traits for different treatments of Fukuhibiki

| Variables | UB | SB | PN | PW | GY | BR | NUtE(g) | NUE (st) | NUtE(st) | NUpE | NUE(g) | Pn(v) | Pn(h) | Pn(gf) | SPAD(v) | SPAD(h) | SPAD (gf) |
| --- | --- | --- | --- | --- | --- | --- | --- | --- | --- | --- | --- | --- | --- | --- | --- | --- | --- |
| UB |  | **0.79** | 0.04 | 0.23 | 0.24 | 0.47 | -0.04 | **0.79** | -0.05 | **0.62** | 0.24 | 0.41 | 0.49 | 0.39 | 0.24 | 0.47 | **0.55** |
| SB |  |  | 0.00 | -0.28 | -0.11 | 0.06 | -0.21 | **1.00** | -0.21 | **0.85** | -0.11 | 0.39 | 0.44 | 0.38 | 0.19 | **0.65** | 0.35 |
| PN |  |  |  | -0.10 | 0.04 | -0.38 | 0.17 | 0.00 | 0.17 | 0.04 | 0.04 | 0.21 | -0.08 | 0.15 | -0.40 | -0.41 | -0.29 |
| PW |  |  |  |  | **0.77** | **0.80** | 0.39 | -0.28 | 0.39 | -0.40 | **0.77** | -0.08 | 0.22 | 0.13 | -0.08 | -0.07 | 0.27 |
| GY |  |  |  |  |  | **0.61** | 0.12 | -0.11 | 0.14 | -0.14 | **1.00** | -0.06 | 0.36 | -0.07 | -0.24 | -0.16 | -0.02 |
| BR |  |  |  |  |  |  | 0.14 | 0.06 | 0.15 | -0.06 | **0.61** | 0.03 | 0.43 | 0.27 | 0.23 | 0.31 | 0.48 |
| NUtE(g) |  |  |  |  |  |  |  | -0.21 | **1.00** | **-0.60** | 0.12 | 0.23 | -0.07 | 0.36 | -0.30 | 0.06 | 0.32 |
| NUE (st) |  |  |  |  |  |  |  |  | -0.21 | **0.85** | -0.11 | 0.39 | 0.44 | 0.38 | 0.19 | **0.65** | 0.35 |
| NUtE(st) |  |  |  |  |  |  |  |  |  | **-0.60** | 0.14 | 0.21 | -0.06 | 0.38 | -0.32 | 0.06 | 0.30 |
| NUpE |  |  |  |  |  |  |  |  |  |  | -0.14 | 0.37 | 0.35 | 0.16 | 0.30 | 0.50 | 0.14 |
| NUE(g) |  |  |  |  |  |  |  |  |  |  |  | -0.06 | 0.36 | -0.07 | -0.24 | -0.16 | -0.02 |
| Pn(v) |  |  |  |  |  |  |  |  |  |  |  |  | 0.13 | 0.30 | 0.44 | 0.41 | **0.57** |
| Pn(h) |  |  |  |  |  |  |  |  |  |  |  |  |  | 0.24 | -0.10 | 0.45 | 0.15 |
| Pn(gf) |  |  |  |  |  |  |  |  |  |  |  |  |  |  | 0.08 | **0.63** | **0.54** |
| SPAD(v) |  |  |  |  |  |  |  |  |  |  |  |  |  |  |  | 0.31 | **0.50** |
| SPAD(h) |  |  |  |  |  |  |  |  |  |  |  |  |  |  |  |  | **0.65** |
| SPAD (gf) |  |  |  |  |  |  |  |  |  |  |  |  |  |  |  |  |  |

Values in bold are different from 0 with a significance level alpha=0.05 and short initial letters for plant traits are described in Fig. 3
