# Supplementary material for: Influence of rice-husk biochar and Bacillus pumilus strain TUAT-1 on yield, biomass production, and nutrient uptake in two forage rice genotypes
Source: PLoS One. 2019 Jul 31;14(7):e0220236. doi: 10.1371/journal.pone.0220236 (PMC6668810; doi:10.1371/journal.pone.0220236)
Supplement: S5 Table — (DOCX) [file pone.0220236.s005.docx]

S5 Table. Correlation matrix (Pearson (n) for grain yield and its related traits for different treatments of LTAT-29

| Variables | UB | SB | PN | PW | GY | BR | NUtE(g) | NUE (st) | NUtE(st) | NUpE | NUE(g) | Pn(v) | Pn(h) | Pn(gf) | SPAD(v) | SPAD(h) | SPAD (gf) |
| --- | --- | --- | --- | --- | --- | --- | --- | --- | --- | --- | --- | --- | --- | --- | --- | --- | --- |
| UB |  | **0.69** | **-0.64** | **0.63** | **0.63** | **0.55** | **0.66** | **0.69** | **0.66** | 0.22 | **0.63** | 0.20 | **0.74** | 0.06 | 0.14 | **0.67** | 0.14 |
| SB |  |  | -0.20 | -0.13 | 0.08 | -0.22 | 0.30 | **1.00** | 0.28 | **0.74** | 0.08 | 0.06 | 0.29 | 0.12 | -0.17 | 0.35 | 0.30 |
| PN |  |  |  | **-0.65** | **-0.71** | **-0.63** | -0.42 | -0.20 | -0.43 | 0.19 | **-0.71** | -0.19 | **-0.59** | 0.09 | -0.03 | **-0.65** | 0.15 |
| PW |  |  |  |  | **0.77** | **0.99** | **0.59** | -0.13 | **0.60** | -0.49 | **0.77** | 0.22 | **0.69** | -0.04 | 0.37 | **0.55** | -0.13 |
| GY |  |  |  |  |  | **0.73** | 0.47 | 0.08 | 0.46 | -0.24 | **1.00** | -0.04 | **0.70** | -0.14 | 0.31 | **0.63** | -0.03 |
| BR |  |  |  |  |  |  | **0.55** | -0.22 | **0.56** | **-0.55** | **0.73** | 0.21 | **0.63** | -0.08 | 0.31 | **0.50** | -0.10 |
| NUtE(g) |  |  |  |  |  |  |  | 0.30 | **1.00** | -0.32 | 0.47 | 0.46 | **0.66** | -0.23 | 0.42 | 0.33 | -0.14 |
| NUE (st) |  |  |  |  |  |  |  |  | 0.28 | **0.74** | 0.08 | 0.06 | 0.29 | 0.12 | -0.17 | 0.35 | 0.30 |
| NUtE(st) |  |  |  |  |  |  |  |  |  | -0.33 | 0.46 | 0.48 | **0.66** | -0.24 | 0.41 | 0.33 | -0.15 |
| NUpE |  |  |  |  |  |  |  |  |  |  | -0.24 | -0.13 | -0.11 | 0.22 | -0.19 | 0.02 | 0.37 |
| NUE(g) |  |  |  |  |  |  |  |  |  |  |  | -0.04 | **0.70** | -0.14 | 0.31 | **0.63** | -0.03 |
| Pn(v) |  |  |  |  |  |  |  |  |  |  |  |  | 0.08 | -0.43 | 0.10 | -0.17 | -0.37 |
| Pn(h) |  |  |  |  |  |  |  |  |  |  |  |  |  | 0.19 | 0.49 | **0.51** | -0.06 |
| Pn(gf) |  |  |  |  |  |  |  |  |  |  |  |  |  |  | 0.05 | 0.12 | -0.09 |
| SPAD(v) |  |  |  |  |  |  |  |  |  |  |  |  |  |  |  | -0.02 | -0.31 |
| SPAD(h) |  |  |  |  |  |  |  |  |  |  |  |  |  |  |  |  | 0.38 |
| SPAD (gf) |  |  |  |  |  |  |  |  |  |  |  |  |  |  |  |  |  |

Values in bold are different from 0 with a significance level alpha=0.05 and short initial letters for plant traits are described in Fig. 3
